# Whole-Genome SNP Analysis Identifies Putative *Mycobacterium bovis* Transmission Clusters in Livestock and Wildlife in Catalonia, Spain

**DOI:** 10.3390/microorganisms9081629

**Published:** 2021-07-30

**Authors:** Claudia Perea, Giovanna Ciaravino, Tod Stuber, Tyler C. Thacker, Suelee Robbe-Austerman, Alberto Allepuz, Bernat Pérez de Val

**Affiliations:** 1 National Veterinary Services Laboratories, U.S. Department of Agriculture, Animal and Plant Health Inspection Service, Veterinary Services, Ames, IA 50010, USA; tod.p.stuber@usda.gov (T.S.); tyler.thacker@usda.gov (T.C.T.); suelee.robbe-austerman@usda.gov (S.R.-A.); 2 Departament de Sanitat i Anatomia Animals, Universitat Autònoma de Barcelona, 08193 Bellaterra, Spain; Giovanna.Ciaravino@uab.cat (G.C.); alberto.allepuz@uab.cat (A.A.); 3 IRTA, Centre de Recerca en Sanitat Animal (CReSA, IRTA-UAB), 08197 Bellaterra, Spain; bernat.perez@irta.cat; 4 OIE Collaborating Centre for the Research and Control of Emerging and Re-Emerging Swine Diseases in Europe (IRTA-CReSA), 08193 Bellaterra, Spain

**Keywords:** whole-genome sequencing, SNP, *Mycobacterium bovis*, bovine tuberculosis, Catalonia, northeastern Spain

## Abstract

The high-resolution WGS analyses of MTBC strains have provided useful insight for determining sources of infection for animal tuberculosis. In Spain, tuberculosis in livestock is caused by *Mycobacterium bovis* and *Mycobacterium caprae*, where wildlife reservoirs play an important role. We analyzed a set of 125 *M. bovis* isolates obtained from livestock and wildlife from Catalonia to investigate strain diversity and identify possible sources and/or causes of infection. Whole-genome SNP profiles were used for phylogenetic reconstruction and pairwise SNP distance analysis. Additionally, SNPs were investigated to identify virulence and antimicrobial resistance factors to investigate clade-specific associations. Putative transmission clusters (≤12 SNPs) were identified, and associated epidemiological metadata were used to determine possible explanatory factors for transmission. *M. bovis* distribution was heterogeneous, with 7 major clades and 21 putative transmission clusters. In order of importance, the explanatory factors associated were proximity and neighborhood, residual infection, livestock-wildlife interaction, shared pasture, and movement. Genes related to lipid transport and metabolism showed the highest number of SNPs. All isolates were pyrazinamide resistant, and five were additionally resistant to isoniazid, but no clade-specific associations could be determined. Our findings highlight the importance of high-resolution molecular surveillance to monitor bovine tuberculosis dynamics in a low-prevalence setting.

## 1. Introduction

*Mycobacterium bovis*, a member of the *Mycobacterium tuberculosis* complex (MTBC), is responsible for causing tuberculosis (TB) in livestock, wildlife, and humans throughout the world. Currently, a few countries have achieved null to the extremely low prevalence of bovine TB (≤0.1%) through strict disease surveillance and preventive measures carried out in accordance with national bovine TB eradication programs [1,2,3,4,5]. In Spain, herd prevalence was 1.9% in 2019. However, at the regional level, the situation is very heterogeneous. The southwestern regions showed the highest values, with a range between 6.6% and 14.9%, while in the northern regions, the range was between 0% and 1.4%. In Catalonia (northeastern Spain), herd prevalence was 0.04% [6,7].

An essential step in elucidating the dynamics of disease is strain characterization, as it sets the framework for identifying possible sources and introductions of infection. Additionally, the degree of genetic diversity within a region may provide insights into the level of disease persistence and spread [8,9]. Previously, studies in Spain have used traditional molecular tools for *M. bovis* genotyping, such as direct variable repeat spacer oligonucleotide typing (DVR-spoligotyping) and mycobacterial interspersed repetitive unit-variable number tandem repeats (MIRU-VNTR) [8,10]. These have successfully captured the broad genetic diversity of this pathogen from various hosts and different regions. Generally, spoligotyping has revealed high *M. bovis* genetic diversity throughout Spain, yielding 265 spoligotype patterns, where SB0121 was the most frequent. More in-depth analysis showed that within SB0121, the loss of spacer 21 is a dominant feature among Spanish isolates [11]. Later, MIRU-VNTR analysis of this dominant genotype resulted in 55 different profiles, highlighting the limited discriminatory power of DVR-spoligotyping and demonstrating the clonal expansion that this group of closely related strains underwent [12].

On a broader phylogenetic scale, four main clonal complexes have been defined for *M. bovis* based on distinct spoligotype signatures, unique genomic deletions, and specific single nucleotide polymorphisms (SNP): European 1 (Eu1) [13], European 2 (Eu2) [14], African 1 (Af1) [15] and African 2 (Af2) [16]. Recently, the addition of clonal complex European 3 (Eu3) has been proposed [17]. *M. bovis* in the Iberian Peninsula, which represents Spain and Portugal, has been characterized by strains mainly within clonal complex Eu2, though Eu1 strains can be found at low frequency [18]. The genomic signatures for Eu2 are the lack of spacer 21 in its spoligotype pattern and a single nucleotide polymorphism (SNP) in guaA [14]. In addition, previous studies in Spain based on data retrieved from epidemiological questionnaires and spoligotype patterns have identified the main causes of herd breakdowns as: residual infections (i.e., persistence of the mycobacteria within the herd), interaction with wildlife reservoirs such as wild boars (*Sus scrofa*), red deer (*Cervus elephus*), or fallow deer (*Dama dama*) and movements of cattle [19,20,21,22,23].

Whole-genome sequencing (WGS) has produced outstanding results for strain characterization and outbreak investigation thanks to its superior discriminatory power [9,24,25]. Recently, WGS has been useful at describing the population structure of *M. bovis* in France and Germany [26,27], contributed to determining sources of *M. bovis* infections in New Zealand and the United Kingdom [25,28], and precisely described the molecular epidemiology of *M. bovis* TB-affected cattle herds in the United States [29]. In Spain, WGS has already been used for investigating the transmission of *M. caprae* between wild boar and goats, revealing the active role of the wild boar as a TB reservoir [30].

TB research in Spain has addressed several issues concerning the situation of bovine TB in the country, such as surveillance sensitivity and strategies, risk factors for herd breakdowns, variability in transmission of TB, the role of wildlife as TB reservoirs, and the influence of non-biological factors in TB detection and control [19,21,31,32,33,34,35,36]. To complement this, the objective of the present study is to characterize *M. bovis* isolates obtained from livestock and wildlife in Catalonia using WGS and SNP analysis to identify epidemiological clusters, as well as to investigate how the isolates from this region fit into the broader global phylogenetic structure.

## 2. Materials and Methods

### 2.1. Sampling

Tissue samples from livestock and wild ungulates were obtained from different species (TB eradication campaigns based on test and slaughter of positive animals, surveillance at abattoirs, and hunting) throughout Catalonia (Northeastern Spain) from 2008 to 2018. Samples were submitted through the Slaughterhouse Support Network (www.cresa.cat/blogs/sesc, accessed on 29 July 2021) to the laboratory at IRTA-CReSA (Bellaterra, Barcelona) for TB investigation. The tissue samples that are submitted to the laboratory include mainly lymph nodes of the head, thorax, and abdomen (such as superficial cervical, parotid, retropharyngeal, tracheobronchial, mandibular, mediastinal, and hepatic), and the lungs and liver.

### 2.2. MTBC Isolation and Identification

**Figure 1:**
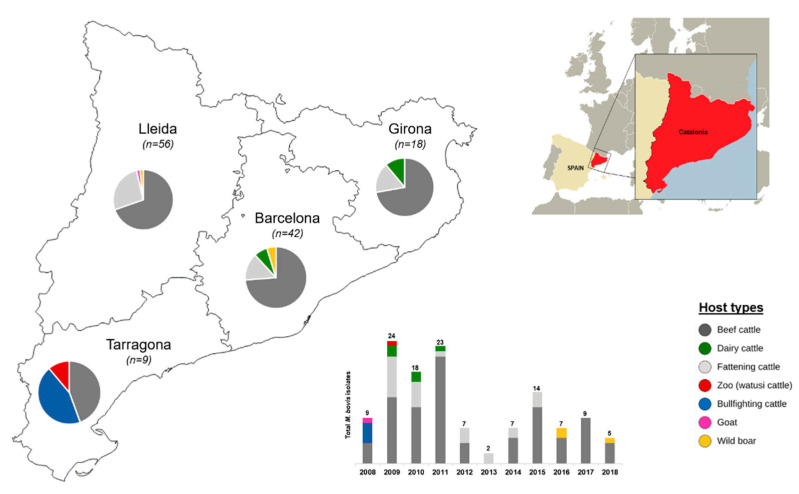
Map of Catalonia showing the origin of the *M. bovis* isolates with respect to geographic region (province) and host type. The stacked bar chart represents the number of isolates that correspond to each year of isolation (2008–2018). Total number of isolates per region and year are indicated, as well as the proportion of isolates for each host type.

Tissues were homogenized in 10 mL sterile water using a Masticator (IUL Instruments, Barcelona, Spain). Next, homogenates were processed two different ways: (a) they were decontaminated for 30 min with a final concentration of 0.35% *w*/*v* hexadecylpyridinium chloride and cultured on solid media (Löwenstein-Jensen with pyruvate and Coletsos, BD Diagnostics, Sparks Glencoe, MD, USA) as previously described [37], or (b) decontaminated with oxalic acid at 5% *w*/*v* for 30 min, neutralized with 1 M NaOH, centrifugated at 2451× *g* for 30 min, supernatants were discarded, pellets were suspended in 1 mL sterile PBS and 0.5 mL of the suspension was added to BBL MGIT tubes (BD Diagnostics) and incubated in BACTEC MGIT 320 system (BD Diagnostics). DNA from 125 positive cultures (from 121 cattle, 1 goat and 3 wild boars) were extracted by boiling bacterial growth for 10 min, and MTBC was confirmed by multiplex PCR as previously described [38]. MTBC confirmed isolates were sub-cultured on 7H11 plates (BD Diagnostics) for 28 days at 37 ± 1 °C, growth was suspended in 1 mL PBS and inactivated at 100 °C for 30 min. Heat-inactivated cells were sent to the United States Department of Agriculture’s National Veterinary Services Laboratories (NVSL) in Ames, Iowa, USA, for WGS. With respect to geographic origin, the *M. bovis* isolates obtained in this study were from the 4 provinces of Catalonia: Barcelona (*n* = 42), Girona (*n* = 18), Lleida (*n* = 56) and Tarragona (*n* = 9) (Figure 1). The number of isolates per year was: 2008 = 9, 2009 = 24, 2010 = 18, 2011 = 23, 2012 = 7, 2013 = 2, 2014 = 7, 2015 = 14, 2016 = 7, 2017 = 9, and 2018 = 5. Finally, isolates were obtained from seven different host types, including beef cattle (BC = 89), fattening cattle (FC = 22), dairy cattle (DC = 5), bullfighting cattle (BfC = 4), zoological-watusi cattle (ZwC = 1), goat (G = 1), and wild boar (WB = 3).

### 2.3. DNA Extraction

At NVSL, genomic DNA extraction was performed using a KingFisher Flex Purification System (Thermo Fisher, Waltham, MA, USA) and the MagMax CORE (Thermo Fisher, Waltham, MA, USA) total nucleic acid isolation kit according to manufacturer’s instructions. For sequencing, at least 20 µL (≥5 ng/µL) of double-stranded DNA was required.

### 2.4. Whole-Genome Sequencing and SNP Analysis

Libraries were prepared using the Nextera XT Kit (Illumina, Inc., San Diego, CA, USA), and sequencing was performed on an Illumina MiSeq device using 2 × 250 paired-end chemistry. Multiple isolates were indexed per lane, providing approximately 50–100× coverage per isolate. Raw sequences were aligned to the reference genome AF2122/97 (GenBank accession NC_002945.4) and SNPs identified using vSNP (see https://github.com/USDA-VS/vSNP, accessed on 29 July 2021, for bioinformatics scripts and Supplementary File S1 for sequencing statistics). Briefly, the alignment was performed using Burrows-Wheeler Aligner (BWA) [39], and SNPs were called using Freebayes [40]. Sites that fell within proline-glutamate (PE) and proline-proline-glutamate (PPE)-polymorphic CG-repetitive sequences (PGRS) were filtered and excluded, as well as SNP positions with a phred-scaled quality (QUAL) score for the alternate nonreference allele lower than 150 or when all positions in a data set had an allele count (AC) equal to 1 when analyzed as a diploid. Integrated genomics viewer (IGV) [41] was used to visually validate SNPs, and SNPs with mapping issues or alignment problems were manually filtered. Phylogenies were constructed with RAxML [42] using the aligned whole-genome SNP sequences under a GTR-CAT model of substitution and a maximum-likelihood algorithm. Tree visualization, annotation, and editing were performed with FigTree (https://github.com/rambaut/figtree/releases, accessed on 29 July 2021) and iTOL (https://itol.embl.de/, accessed on 29 July 2021) [43]. As the output from the vSNP pipeline, SNP tables for each major clade were generated; these are formatted Excel tables that group and sort isolates and SNP according to relatedness and reflect exactly what is shown by the phylogenetic tree, which provides transparency of the results. In the SNP tables, the columns identify the genome location of the SNP calls, and the isolates are listed in the rows. The reference (*M. bovis* AF2122/97, NC_002945.4) is listed across the top and is identified as the “reference call.” All SNP are highlighted. Map quality for each SNP is indicated and is an average of the map quality scores of each isolate at that position. A score of 60 is the highest possible. The annotation of each SNP is listed at the bottom of the SNP table, indicating product, gene name and locus tag. Finally, minimum spanning trees were constructed using PHYLOViZ [44] with default parameters using the concatenated SNP matrices for each of the *M. bovis* groups as input.

Additionally, the sequences from Catalonia were compared against a global set of 637 *M. bovis* isolates obtained from public repositories (https://www.ncbi.nlm.nih.gov/sra/, accessed on 29 July 2021). From previous publications regarding WGS analysis of *M. bovis*, a search was performed in NCBI’s Sequence Read Archive to obtain the sequences. Supplementary File S2 contains the list of accessions for the global data set used in this analysis and provides information such as year of isolation, host, and country of origin, which was obtained from the publications associated with the sequences or through direct contact with the submitters.

### 2.5. Characterization of Antibiotic-Resistant Mutations and SNP on Virulence Genes

Both raw and assembled data (using SPAdes) were analyzed for known antimicrobial resistance mutations using PointFinder and the *Mycobacterium tuberculosis* database for comparison [45]. SNPs on known virulence genes were investigated to identify possible virulence traits associated with the different phylogenetic clades of *M. bovis* from Catalonia. We used a previously compiled list of genes [26] to investigate the annotations associated with the SNPs obtained from vSNP and analyzed the distribution of the most frequent categories among the clades.

### 2.6. In Silico DVR-Spoligotyping

*M. bovis* spoligotypes were obtained through the vSNP pipeline with the “spoligo” function. This function outputs a text file that lists the read counts for each of the spacer regions and calculated the BIN code. The BIN code was cross-referenced with the Mbovis.org database (www.mbovis.org, accessed on 29 July 2021) to obtain the SB number (Supplementary File S1).

### 2.7. Epidemiological Associations

A threshold of 12 distinct SNP positions was used to identify strains possibly involved in transmission events, similar to what has been previously proposed [46]. Briefly, the vSNP pipeline produces SNP matrices as Excel tables that group and sort the isolates and SNPs according to evolutionary relationships (genetic relatedness), which are shown in Supplementary File S3. These were used to manually inspect SNP profiles. Based on patterns observed in the data, SNP distances were calculated, and isolates that were within a pairwise distance of 12 SNPs from each other were considered a “putative transmission cluster”. In order to assess the most likely cause of a transmission cluster, we investigated the epidemiological associations between the animals involved and classified them within six most likely explanatory factors: (1) residual infection (same herd), (2) same municipality (neighborhood), (3) same county (proximity), (4) farm-to-farm movements (movement), (5) shared pasture, and (6) livestock-wildlife interaction. When the information was not enough to pinpoint a specific explanatory factor, it was defined as unknown.

#### Animal Movements

To investigate possible points of transmission or movement links between animals involved in a putative transmission cluster, movement records were analyzed for each of the animals involved. As a whole, records spanned a 20-year window (1999–2018), but for each animal, this varied from 1 to 14 years. For each animal, movement records included for analysis were: farm of origin, secondary farms they were transferred to, and communal grazing areas. These were defined as destinations, and when at least two animals in a cluster shared a destination, this was defined as a movement link. Additionally, animal movements were classified into four main categories: movement inside Catalonia (MIC), introduction from outside of Spain into Catalonia (IOSC), introduction from other regions of Spain into Catalonia (ISC), and movement from Catalonia to other regions of Spain (MCS).

## 3. Results

### 3.1. Spoligotyping

In silico spoligotyping identified a total of 27 spoligotypes, all registered in the international database Mbovis.org (Table 1). Of these, almost half (12/26) were orphan spoligotypes. The three most frequent spoligotypes were SB0121 (30/125; 24%), SB0134 (20/125; 16%), and SB1337 (14/125; 11.2%). More specifically, SB0134 was the most frequent in fattening cattle and was found in only one other host type-beef cattle. SB0121 was the most widely distributed across host types, absent only from bullfighting cattle and Watusi. SB0339, which was recovered from the Watusi, was not found in any other host. Three spoligotypes were found in wild boar (SB0121, SB0119, and SB1337); interestingly, SB0119 and SB1337 were identified in one other host type, beef cattle. Additionally, the spoligotypes obtained from bullfighting cattle (SB1685, SB1095, and SB1192) were exclusive to this host type. All of the spoligotypes identified in dairy cattle were also found in beef cattle. Finally, for the fattening cattle, four spoligotypes (SB2312, SB0130, SB0142, and SB1259) were not recovered from any other cattle host type.

### 3.2. WGS-Based Phylogenetic Structure of MTBC Isolates from Catalonia

A total of 125 *M. bovis* genomes were obtained, with an average of 635.5 SNP sites (range of 168 to 834) per genome and a final SNP alignment length of genomes of 5049 informative SNP sites (Supplementary File S3). Sequencing statistics for these isolates can be consulted in Supplementary File S1. For the phylogenetic analysis, a total of 182 sequences were included, 125 *M. bovis* from this study and 52 *M. caprae* sequences from a previous study [30], as well as *M. bovis* reference strains (*M. bovis* AF2122/97-NC_002945.4, *M. bovis* Ravenel-SRR3135072, *M. bovis* BCG-SRR5485728 and *M. bovis* AN5-SRR3135071). Overall, seven major clades were identified by WGS for the *M. bovis* isolates obtained from livestock and wildlife in Catalonia, Spain, plus the *M. caprae* clade. These clades, or major phylogenetic groups, have been previously reported [29]. Defining SNP characteristics for these groups are described in Table 2.

**Figure 2:**
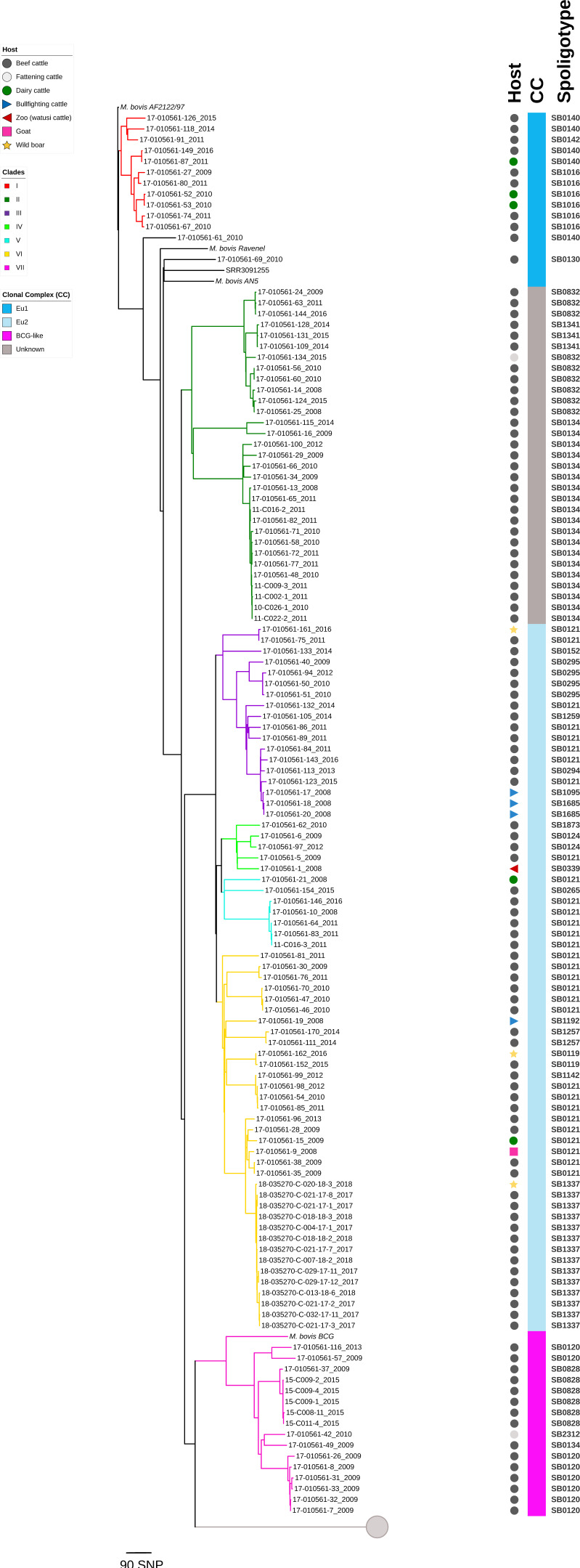
High-resolution maximum-likelihood phylogenetic tree including 125 *M. bovis* and 52 *M. caprae* isolates recovered from livestock and wildlife in Catalonia, Spain, between 2008 and 2018. Clades, clonal complexes, and host type are identified according to the legend. Isolate tags include isolate ID and year of isolation. The spoligotypes for all isolates are given. Displayed in the columns on the right. The *M. caprae* clade is collapsed (gray circle). *M. bovis* reference strains (*M. bovis* AF2122/97, *M. bovis* Ravenel, *M. bovis* AN5, and *M. bovis* BCG) are included for perspective. The scale bar represents a branch length of 90 SNPs.

Furthermore, two isolates (17-010561-69 and 17-010561-61) fell on separate, single branches, possibly indicating an additional two clades that may be part of the full genetic diversity of *M. bovis* in Catalonia. All clades, except one, fall within the known *M. bovis* clonal complexes (CC), including Eu1, Eu2, and the “BCG-like” clade derived from the ancestral SB0120 spoligotype. The major clades, with their corresponding clonal complex, and spoligotypes are labeled I through VII in Figure 2. An SNP table with the overall SNP alignment for all the clades is shown in Supplementary File S3.

In general, the phylogenetic structure of *M. bovis* isolates from Catalonia did not show any specific pattern, such as grouping by host, year of isolation, or geographic region. The largest was Clade VI, with 35/125 of isolates, and it contained the widest variety of hosts (dairy, beef and bullfighting cattle, goat, and wild boars). The spoligotypes associated with isolates in this clade were mostly SB0121 and SB1337; others included SB0119, SB1257, and SB1192. Clade II followed closely with 31 isolates, mostly obtained from beef/fattening cattle, and the associated spoligotypes were SB0832, SB0134, and SB1341. Clades III and VII contained 18 and 16 isolates, respectively. Spoligotypes SB0121, SB0295, SB1685, SB0294, SB1095, SB1259, and SB0152 were associated with Clade III, while Clade VII had spoligotypes SB0120, SB0134, SB2312, and SB0828. Finally, Clades I, IV, and V included 11, 5, and 7 isolates, respectively. Spoligotypes SB1016 and SB0140 were predominant in Clade I; Clade IV included SB1873, SB0124, SB0339, and SB0121; and SB0121 was predominant in Clade V. Additionally, spoligotype SB0134 was associated with two clades (II and VII), which is inherent to the homoplastic nature of spoligotyping, and SB0121 spanned across four clades (III–VI), typical of the Eu2 complex. With respect to *M. bovis* clonal complexes, Clades III–VI corresponded to Eu2, Clade I to Eu1, and Clade VII to the “BCG-like” clade. Clade II did not correspond to any of the presently known complexes.

### 3.3. Epidemiological Investigations and Putative Transmission Clusters

Within each of the main clades, clusters of closely related isolates (≤12 SNPs) were labeled as a subgroup of the main genetic clade to which they belonged (i.e., I.1, II.2, III.4, etc.). A total of 21 clusters were identified, containing between 2 and 14 isolates, for a total of 83 isolates in all clusters (Table 3). More than half of the clusters (12/21) involved only beef cattle, three involved wildlife (wild boar) and beef cattle, three involved imported beef cattle from outside of Spain, and a couple involved dairy and beef cattle. The average SNP distance between the isolates involved in a cluster ranged from 0 to 7 SNPs. Additionally, eight of the clusters involved isolates recovered within the same year, nine involved isolates from two different years, and four involved isolates from three years. In three of the clusters (II.2, II.4, and V.1), isolates were recovered at least seven years apart, indicative of persistent bovine TB infection. Furthermore, analysis of the movements for the animals involved in a putative transmission cluster resulted in all but four having shared at least one destination (movement link), it being the farm of origin, a secondary farm, or a communal grazing area (Table 3). Interestingly, four of the clusters involved animals with no observable movement links, despite being involved in movements within Catalonia (MIC; I.2, I.3, II.2, and VII.3). In addition, the majority (18/21) involved animals that had movements only inside Catalonia (MIC), and three involved animals that were introduced into Catalonia from outside of Spain (IOSC).

**Figure 3:**
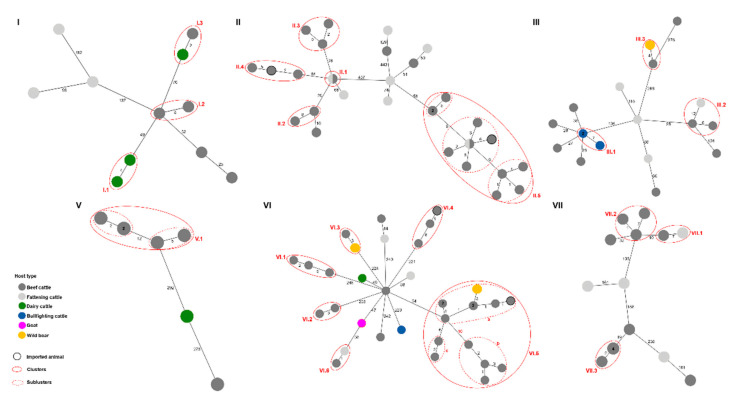
Minimum spanning trees (MSTs) of the putative transmission clusters identified within the main WGS clades (I–III and V–VII) based on SNP analysis of 125 *M. bovis* isolates obtained in Catalonia, Spain, from 2008 to 2018. Nodes are colored according to host type. Clusters (solid red circles) and subclusters (dashed red circles) are identified. The nodes that have a black outline represent cattle imported into Catalonia from outside of Spain. Numbers inside nodes indicate the total number of isolates represented by that node. The numbers on the branches connecting the nodes represent the number of segregating SNP sites between a pair of nodes. MSTs were constructed using the whole-genome concatenated SNP sequences for each main clade and PHYLOViZ2.0 (https://online.phyloviz.net/index, accessed 29 July 2021).

For nearly all clusters that involved the same host type, putative transmission occurred mainly between beef cattle (19/21 clusters), as this host type was the most common. Dairy and bullfighting cattle were only involved in two and one clusters, respectively (Figure 3). More specifically, regarding clusters where beef cattle were involved, in 10 of 19 clusters, transmission occurred exclusively between beef cattle; in 5 of 19 clusters, it was between beef and fattening cattle (II.1, II.5, III.2, VI.6, and VII.1); in 3 of 19 clusters transmission involved beef cattle and wild boar (III.3., VI.3, and VI.5); and finally, only one cluster involved beef and dairy cattle. Regarding the clusters with the participation of wild boars, in cluster III.3, the wild boar isolate accumulated 4 SNPs since sharing a common ancestor with the beef cattle isolate, which actually matched the ancestral genotype (Figure 3, Supplementary File S3). In cluster VI.3, the beef isolate accumulated 3 SNPs since sharing a most recent common ancestor (MRCA) with the wild boar isolate, which in this case matched the ancestral genotype (Figure 3, Supplementary File S3). Furthermore, in cluster VI.5, which includes a total of 14 isolates, the wild boar isolate accumulated 3 SNPs since its MRCA with the beef cattle isolates; in this case, the cattle isolates matched the ancestral genotype (Supplementary File S3). The beef cattle involved in these clusters had movements that implicated communal grazing areas, which could represent a source of infection for these two host types (BC ↔ WB). Additionally, one cluster involved putative transmission between dairy and beef cattle (I.3); here, only two additional SNPs differentiate the beef isolate from the dairy isolate (ancestral genotype); however, no movement links for these animals could be determined with the available information. Finally, four clusters involved at least one imported animal (II.4, II.5, VI.4, and VI.5); for these, the Catalonia beef cattle isolates showed the ancestral genotypes (Supplementary File S3). Furthermore, upon closer analysis, three clusters showed subclusters of isolates that had a ≤4 SNP difference: II.5 (II.5a and b), V.1 (V.1a and b), and VI.5 (VI.5a, b, and c) (Figure 3, Supplementary File S3).

Table 4 summarizes the epidemiological associations that could be determined for the putative transmission clusters. These included six most likely explanatory factors, such as residual infection (same herd), neighborhood/municipality of origin (neighborhood), county of origin (proximity), farm-to-farm movements (movement), communal grazing area (shared pasture), and livestock-wildlife interaction. When the information was not enough to pinpoint a specific explanatory factor, it was defined as unknown. The distribution of the explanatory factors across the different putative transmission clusters was heterogeneous, as no one explanatory factor was more prevalent than the rest for any of the clusters, and each cluster had at least one explanatory factor. The most obvious explanatory factor, residual infection, accounted for four clusters (I.1, III.1, V.1a, and VI.2). For cluster I.3, which involved putative transmission between dairy and beef cattle, the only possible explanatory factor that could be deduced from the available information was proximity. All clusters that involved wild boar were classified within the “livestock-wildlife interaction” factor. Due to the different subclusters, cluster VI.5 included several explanatory factors (neighborhood, shared pasture, and livestock-wildlife interaction). Finally, for clusters I.2 and II.2, which involved beef cattle, no explanatory factors could be determined from this data set.

### 3.4. Cross-Sectional Data Analysis in a World Context

**Figure 4:**
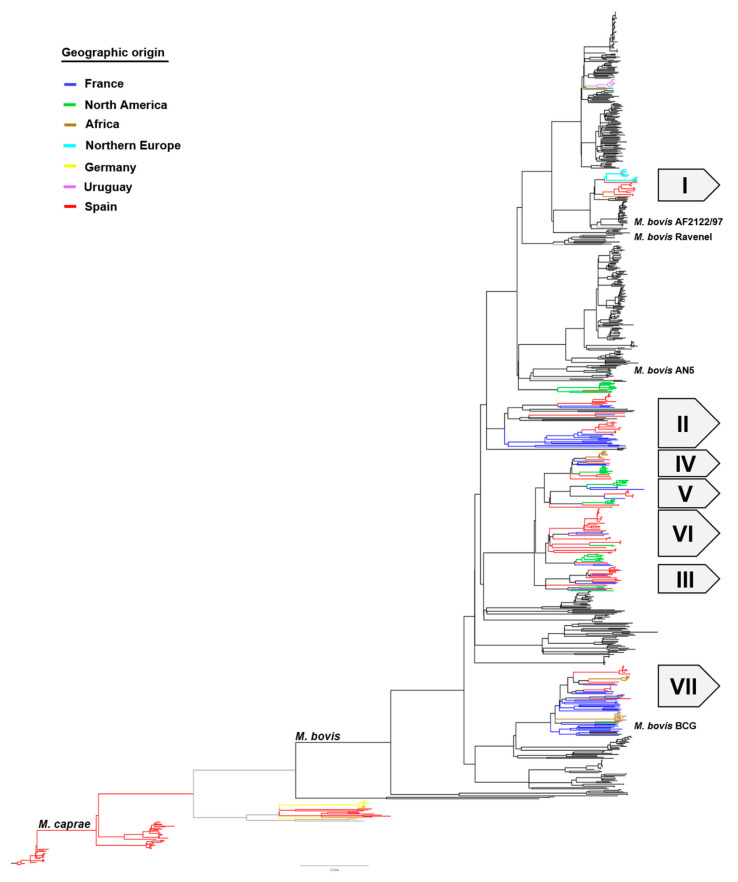
Global phylogenetic structure of *M. bovis* isolates representing a worldwide data set. *M. caprae* is used as the outgroup. The main WGS clades identified for Catalonia are indicated on the right (I–VII). The geographic origin of the isolates is indicated by the branch colors according to the color key (France—blue, North America—green, Africa—brown, Norther Europe—cyan, Germany—yellow, Uruguay—purple, Catalonia—red). Reference isolates for *M. bovis* (AF2122/97, AN5, Ravenel, BCG) are included for perspective. The scale bar represents substitutions per site. An expanded version of this tree is shown in Supplementary File S5.

A comparative analysis was performed including 177 sequences from Catalonia (125 *M. bovis* from this study and 52 *M. caprae* from Ciaravino et al., 2020) plus 637 sequences retrieved from the NCBI Sequence Read Archive Database (https://www.ncbi.nlm.nih.gov/sra/, accessed 29 July 2021) (Supplementary File S2), representing 28 countries across North America, South America, the Caribbean, Africa and Europe, as well as reference strains *M. bovis* AF2122/97, *M. bovis* Ravenel, *M. bovis* AN5 and *M. bovis* BCG, revealed possible ancestor origins for the Catalonia isolates (Figure 4).

Overall, the analysis revealed that the isolates from Catalonia are more closely related to isolates from three main regions: Northern Europe (United Kingdom and Ireland), North America (Mexico and the United States), and France. Isolates from Clade I clustered with isolates from the United Kingdom and Ireland. In this clade, one isolate that was recovered from a U.K.-imported animal (fattening) was only 4 SNPs from a shared common ancestor with isolates from Northern Ireland (Supplementary File S4, Clade I). Isolates in Clade II were at least 60 SNPs from sharing a common ancestor with isolates from France. Clade III showed additional relationships to isolates from Mexico. In clade IV, isolates from South Africa are at least 28 SNPs from sharing a common ancestor with an isolate from Catalonia. Though in Clade V there are isolates from France, the United States, and Mexico, their relationship to Catalonia isolates is distant (>75 SNPs), and the same can be said for Clade VI. In Clade VII, however, an isolate recovered from a France-imported animal is 6 SNPs from sharing a common ancestor with a deer isolate from France (Supplementary File S4, Clade VII). This clade corresponds to the “BCG-like” complex, and the French isolates dominate in this clade. Finally, with respect to the *M. caprae* clade, there is one Catalonia cattle isolate that is within 3 SNPs of sharing a common ancestor with a cattle isolate from Germany (Supplementary File S4, *M. caprae*).

### 3.5. Virulence and Antimicrobial Resistance Traits

**Figure 5:**
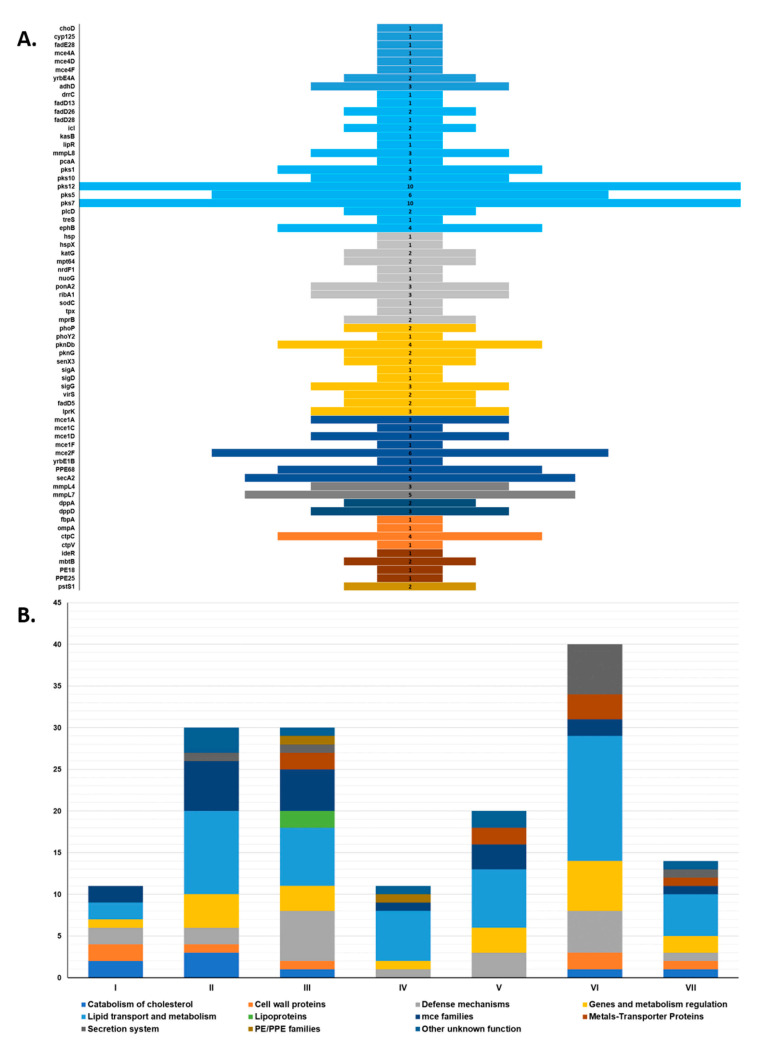
Distribution of virulence-associated SNPs. (**A**) Frequency of SNP-hits among 68 virulence genes; (**B**) within-clade distribution of SNPs according to functional class. A more detailed list of all the SNPs associated with virulence genes can be consulted in Supplementary File S6.

To further investigate the above-mentioned clades, we analyzed the frequency at which SNPs fell within genes to be essential for virulence. A total of 156 SNPs were found to be associated with 68 virulence genes categorized into 11 main functional classes: catabolism of cholesterol, cell wall proteins, defense mechanisms, genes and metabolism regulation, lipid transport and metabolism, lipoproteins, mce families, metals-transporter proteins, secretion system, PE/PPE families, and other unknown functions (Figure 5, Supplementary File S6). Of these, 32 were synonymous and 124 were nonsynonymous. Only Clade III SNPs were associated with all 11 categories. Clade VI had the highest number of SNPs associated with virulence genes (*n* = 40), while Clade I had the least (*n* = 10). Four categories were common to all clades: defense mechanisms, genes and metabolism regulation, mce families, and lipid transport and metabolism. The latter was also the most frequent overall, with a total of 52 SNPs associated with 17 genes related to lipid transport and metabolism (*treS*, *kasB*), mycolic acid synthesis (*pcaA*, *lipR*, *adhD*), synthesis of complex lipids (*pks1*, *pks5*, *pks7*, *pks10*, *pks12*, *fadD26*, *fadD28*, *drrC*, and *mmpL8*), and other genes related in lipid synthesis (*plcD*, *icl*). The least frequent category was lipoproteins, with only two SNPs and one gene associated with it (*pstS1*). Additionally, genes *pks7* and *pks12* presented the most SNP-hits with 10 each, and all polyketide synthase genes accounted for 19% (29/156) of the total virulence-associated SNPs.

With regard to antimicrobial resistance, both methods (raw data and assembled data) produced identical results (Table 5). As is characteristic of *M. bovis*, all of the isolates (125) showed resistance to pyrazinamide due to an SNP change in *pncA* (CAC > GAC) that causes a change of histidine to aspartic acid. Additionally, five isolates also showed resistance to isoniazid by a mutation in a promoter region of *fabG1*.

## 4. Discussion

In order to complement the work performed during a decade (2008–2018) of surveillance, the objective of this study was to use WGS and SNP analysis to characterize the *M. bovis* isolates obtained from livestock and wildlife in Catalonia to identify transmission clusters and use this characterization to determine how the isolates from this region fit into the broader global structure of *M. bovis*.

### 4.1. Isolates’ Demographic Attributes

In terms of the host types analyzed in this study, Catalonia’s livestock sector comprises two main cattle production types: dairy and beef. The latter includes “fattening” cattle as it is a stage in the production cycle of beef cattle; additionally, raising bullfighting cattle is traditional in Spain; however, in Catalonia, it is rare (i.e., approximately 12 out of 5000 herds) [6]. For epidemiological purposes, beef cattle were classified into “fattening” (cattle up to 24 months of age) and “beef” (cattle older than 24 months) and considered as separate host types. From 125 isolates obtained, most were from beef and fattening cattle (88.8%, 111/125). These two host types present a higher TB prevalence compared to dairy cattle, according to the TB surveillance program (2.03% versus 0.71%, respectively) [6]. This may be directly related to cattle population, for which the Catalan cattle census reports ~85% beef cattle, ~15% dairy, and <0.3% bullfighting cattle. In relation to this, sample submission to the lab for TB diagnostics is higher for beef cattle because more field tests are performed due to the need for animal movements (such as the sale of animals or movement to pasture zones), and in the case for reactor animals that are culled and subsequently sampled for laboratory tests. Dairy cattle were only represented by five isolates (4%), which, as mentioned above, may be in relation to the lower prevalence of TB in this production type [6,21,31]. Bullfighting cattle followed with four isolates (3%), and even though it presents the highest prevalence of the three production types (beef, dairy, and bullfighting) [6], Catalonia’s bullfighting cattle population is very small in comparison to beef and dairy, as well as the rest of Spain, accounting for approximately 12 out of 1142 bullfighting cattle-raising farms in the country [6]. As for goats and wild boar (with 1 and 3 isolates, respectively), goats have been found infected mostly with *M. caprae* [30,47,48], while the wild boar has been identified as a reservoir for both *M. bovis* and *M. caprae* [49,50]. Finally, one isolate was recovered from a Watusi animal from a zoological exhibit; there is only one other report of TB in captive Watusi cattle in the scientific literature from a study performed in Mexico [51]. In Africa, in its native setting, the Watusi are commonly used for milk, meat and leather, but in Spain, this breed of cattle is mainly seen in zoological settings.

It is important to mention that the isolates used in this study are a subset of a larger collection of isolates maintained at the IRTA-CReSA laboratory and are meant to represent the population genetic diversity of *M. bovis* in Catalonia, taking into account two main aspects: first, they are representative of the major spoligotyping-VNTR types and adjacent variants and second, they cover different host types, geographic regions (municipalities) and temporal/epidemiological contexts (years of isolation and farms of origin). Briefly, with respect to year of isolation, the total number of isolates per year (2008–2018) reflects the tendency of TB prevalence, with the most isolates seen for years 2008–2011, in which herd TB prevalence was higher in Catalonia (0.59–0.85%), and then decreasing also as the prevalence decreased. A similar scenario was reported in a study in Portugal [52]. However, the same cannot be said for the number of isolates per province and TB prevalence in that region.

### 4.2. Genetic Diversity

A total of 27 spoligotypes are represented by this data set, where SB0121, SB0134, and SB1337 were the three most frequent. Spoligotypes SB0121 and SB0134 are also found in Portugal and France [52,53], reflecting the long trade relationships between these countries; however, for SB1337, reports outside of Spain were not found (www.mbovis.org, accessed on 29 July 2021) and may even be exclusive to Catalonia. Given that this spoligotype is mainly concentrated in the Pyrenees, further studies in Spain and France might provide more insight into this matter. A brief comparison using the profiles reported in the Mbovis.org database showed that 10 of the spoligotypes found here have been reported for Spain, one for Portugal (SB1095), two for France (SB0832 and SB0828), and one for the United Kingdom (SB0142). However, it is possible that not all of the spoligotypes found in these countries are reported within the database, such as SB0140, which is very common in Great Britain [18], so this may be underestimated. In general, spoligotyping and WGS correlated well, though clearly, WGS resolved phylogenetic relationships at a finer scale.

The SNP-based phylogenetic analysis identified seven clades (I–VII). These corresponded to 123/125 of the isolates and may represent the general population structure and genetic diversity of *M. bovis* in Catalonia; however, two additional clades may exist, as two isolates (17-010561-69/SB0130 and 17-010561-61/SB0140) were located on two separate branches that did not cluster with any of the clades identified here. Future studies might benefit from including more isolates with the same spoligotype/VNTR profiles as these to gain more insight into this matter. A correlation of these clades with the main clonal complexes described so far for *M. bovis* indicates that Eu1 and Eu2 are well represented in this data set, as well as the “BCG-like” clonal complex. In Spain, one other study used WGS, in which case an outbreak of *M. caprae* was investigated [30]. In Europe, recent studies in France [26] and Germany [27] also took advantage of this high-resolution technology to delineate the population structure of *M. bovis* in each country, finding 9 and 13 clades, with 87 and 131 *M. bovis* isolates, respectively. In both, Eu1, Eu2, and the “BCG-like” clonal complexes were well represented, with Af1 and Af2 also present in Germany, plus additional clades that do not fall within the defined complexes. In this study, one clade (associated with spoligotypes SB0134, SB0832, and SB1341) did not correspond to any of the known clonal complexes. The authors of [54] identified eight new groups in addition to the known complexes, and of these, Clade II from our study correlates with Unknown 7, which was also found in Germany. According to the aforementioned study, this clade has a geographical range within Western and Southern Europe, as well as Northern and Eastern Africa.

### 4.3. Transmission Clusters

A threshold of 12 SNPs was established to identify strains possibly involved in transmission events, as previous studies have suggested [27,28,29,46]. This threshold resulted in the identification of 21 putative transmission clusters. Half of these included only a pair of isolates, mostly obtained from one host type (beef cattle) and isolated within a three-year period. However, a few involved transmission between different host types, including beef-dairy and beef-wildlife. In a previous study [21], seven possible causes of bovine TB breakdowns were assessed in Spain, including: (1) residual infection, (2) introduction of infected cattle, (3) presence of infected goats, (4) contiguous spread, (5) sharing of pastures, (6) interaction with wildlife, and (7) contact with infected humans. In the present study, the explanatory factors attributed to the putative transmission clusters identified through WGS were (1) residual infection, (2) neighborhood (similar municipality), (3) proximity (similar county), (4) movement, (5) shared pasture, (6) livestock-wildlife interaction, and (7) unknown. For the sake of comparison, “neighborhood” and “proximity” are similar to “contiguous spread”, “movement” is homologous to “introduction of infected cattle”, and “residual infection”, “shared pasture” and “livestock-wildlife interaction” are exactly as previously defined (Table 6). In our study, only two most likely causes were not determined as explanatory factors, “presence of infected goats”, and “contact with infected humans”, which agrees with the results obtained recently by [19]. After an “Unknown” likely cause, a high proportion of bovine TB breakdowns were attributed to “residual infection” by both [21] and [19], while in this study, “contiguous spread” was the most frequent explanatory factor. “Introduction of infected cattle” increased from 5.1% to 13.8%, which is similar to our results of 15%. Finally, the proportion of putative transmission clusters explained by “shared pasture” and “interaction with wildlife”, which were 12% and 15%, respectively in this study, were in agreement with the proportions described by [21] for the same categories. It is interesting how in spite of the different methodologies used for each of the studies, WGS could lead to comparable results. This highlights the fact that while WGS is a powerful tool for investigating sources of infection, an adequate epidemiological investigation must accompany the findings [55]. Some key aspects that could partially contribute to the differences in results obtained by [21], ref. [19] and this study are, first, that this study analyzed data from Catalonia only, while previous studies analyzed data from different autonomous communities, especially with regard to “wildlife interaction” since the importance of these species in the maintenance of bovine TB is heterogeneous across Spain [54,56]; second, in this study, “contiguous spread” encompasses wider criteria, as it also includes farms from the same county, while in the previous studies this only included farms in a 1 km radius.

While these risk factors operate at different scales and may vary across regions, other epidemiological studies have identified a number of risk factors associated with bovine TB herd breakdowns, such as the purchase of cattle, the occurrence of bovine TB in contiguous herds and/or surrounding areas and herd size, and to a lesser degree farm and herd-management practices such as farms having multiple premises, the use of certain housing types, etc. [57,58].

In general, the most consistently identified risk factors are biologically plausible and consistent with known transmission routes involving cattle-cattle (or other livestock) and wildlife-cattle pathways [59,60]. Clusters I.1, III.1, V.1a, and VI.2 showed within-herd transmission, as the animals from each cluster originated from the same herds. Within-herd transmission is the most problematic due to the challenges that can pose certain herd-management practices that do not prevent direct contact between infected and non-infected animals, in addition to contaminated soil, feed, and water from infected herd-mates [60]. Congenital transmission is rare, and transmission via milk is easily preventable by pasteurization [61,62]. Between-herd transmission can be attributed to all clusters in which the most likely explanatory factors were neighborhood, proximity, movement, and shared pasture. For the first two, this could be due to sharing a common boundary or damaged fences that allow for cattle to mix in an uncontrolled way, or even common access with wildlife reservoir [60,63]. With regard to movement, pre-movement testing such as the tuberculin skin test is commonly performed, but the sensitivity of the test may be influenced by the number of animals, as well as the size of the herd of origin, not to mention false-negative results in cases of recent infection where the immune response has not yet developed [35]. As for shared pastures, this practice predisposes contact with other herds, thereby increasing the risk of disease transmission among herds either by animals that inter-mingle, have access to contaminated pasture and soil, and/or infected wildlife [64,65]. Therefore, an important aspect to consider with respect to the potential spread of diseases on communal grazing areas is the level of mixing between animals from different origins. In this regard, [65] showed that the level of mixing might have an effect on bovine TB spread, which in turn may be influenced by herd size [66,67]. More studies are needed to properly estimate the mixing pattern between animals from different herds.

Interestingly, one cluster involved transmission between beef and dairy cattle, which often seems like a rare event since beef and dairy herds are usually raised in separate systems. These isolates were only 2 SNPs apart, which strongly suggests a link between them, and although a direct event of transmission has not been identified, the only link (or explanatory factor) found was “proximity” (i.e., belonged to the same county, <7 km). In a study performed in Northern Ireland, the authors showed borderline significance for spatial proximities of 2 km but none for 5 km [9]. Besides proximity, another possible explanation for the genetic association between these isolates might be that sometimes male-born calves from dairy farms are sold to beef-raising farms, making it possible for transmission to happen between these systems.

For bullfighting cattle, transmission has been attributed to animals from the same herd and/or wildlife that inhabit the premises of such herds [68]. Neither spoligotyping nor WGS could match the bullfighting cattle isolates closely to another host type from this data set. Cluster III.1 involved three isolates, separated by only 7 SNPs, though two had identical SNP profiles. While these were more closely related to isolates from beef cattle, there is a more than 32 SNP pairwise distance, so no clear conclusions could be drawn. Previously, SB0295 was identified as the most prevalent in this host type, but the spoligotypes identified here were SB1095, SB1192, and SB1685, which does not match what was previously reported [69]. Further studies regarding TB in bullfighting cattle may be needed to gain more insight into the epidemiology of the disease in this host type.

The exact origin or direction of infection (i.e., which animal infected which) could not be determined with this data set, but the pairwise SNP distances between isolates (≤12 SNPs) made it possible to follow epidemiological data to identify explanatory factors. WGS provides high-level resolution for strain characterization, which is a powerful tool for building databases against which isolates can be compared to identify closely related strains to understand how disease is introduced into a particular setting or how residual infection persists [25,29].

### 4.4. Catalonia M. bovis in a World Context

Often the comparison of *M. bovis* genotypes from different regions of the world has revealed patterns influenced by the historical trade of cattle [18,70], as well as the natural migration of domesticated livestock [71], and the higher resolution of WGS has provided insight into the evolutionary origins of *M. bovis* as a whole [72,73]. In this study, the comparison of Catalonia *M. bovis* isolates against a global data set showed clustering with isolates from the United Kingdom, France, United States, and Mexico, which agrees with previous reports regarding the distribution of *M. bovis* lineages throughout the world. For example, we found evidence that showed the introduction of a foreign strain as an isolate from U.K.-imported beef cattle matched an isolate from Northern Ireland within 4 SNP. Similarly, two additional imported beef cattle (U.K. and Ireland) also clustered with these isolates. Aside from historical trade, neighboring countries often maintain close commerce relationships due to the ease of transport. For example, the United States and Mexico have a long-standing trade of cattle, which reflects the high degree of shared *M. bovis* genotypes [74]. For Spain, neighboring countries include Portugal and France, and the results are seen here support this regional distribution of genotypes [26,52]. Interestingly, two Catalonia isolates clustered with isolates from South Africa, which may be a function of the Spanish colonization of Africa in the first third of the 20th century [75], or it could go further back to the routes and scattering of cattle in Africa, which subsequently reached Spain [76]. Further studies including a larger set of isolates from different regions of Africa may provide more insight into this matter.

### 4.5. Virulence and Antibiotic Resistance Factors

SNPs were investigated to determine if they fell within genes associated with virulence, and out of 181 genes obtained from a previously compiled list [26], only 68 were found to contain SNPs. After categorizing them into 11 functional classes, genes involved in lipid transport and metabolism had the highest number of SNP-hits. The relationship of lipid and fatty acid metabolism to mycobacteria virulence has been well studied [77], so the high number of SNPs (52 out of 156) associated with this category may be indicative of how constant remodeling of the cell wall by the mycobacteria has allowed for it to adapt to the various hosts it infects and achieve maximum survival. Within this category, genes pks7 and pks12 were also found at a high degree of mutation by [26], with 8 and 49 variants, respectively, while in this study, each presents 10 variants, which were the highest number of mutations found in a single gene. The average number of mutations per gene was 2.3, with most genes having between 1 and 3 mutations. Other categories with a high number (above average) of mutations were defense mechanisms (*n* = 20), genes and metabolism regulation (*n* = 20) and mce families (*n* = 20). Within defense mechanisms, oxidative and nitrosative stress genes had the most mutations, which makes sense given that resistance to macrophage-mediated killing by reactive oxygen/nitrogen species is critical to the virulence of mycobacteria [78]. For genes and metabolism regulation, sigma factors and protein kinases (Pkns) had the most SNP-hits; it has been suggested that crosstalk among Pkns, sigma factors, and two-component systems help the mycobacteria adapt to external stimuli [77]. Finally, the mce family proteins have been shown to confer mycobacteria the ability to enter into mammalian cells (mce = mammalian cell entry) [79], so continuous adaptation of these factors through mutations may be key for propagation. Interestingly, 79.5% of all SNPs were nonsynonymous, posing a greater risk of causing modifications in the structure/function of the final product. Previously, a comparable proportion of nonsynonymous SNPs was obtained by [26] at 69%. While these are only predicted mutations, further investigations on protein structure/chemistry, secreted proteins, cell wall antigens, transmembrane proteins are needed to fully understand the effect of these mutations on phenotype and thus pinpoint specific differences across the main *M. bovis* groups.

In this respect, Clade VI presented by far the higher number of variants than the other clades (Figure 5B), and most of this increase was represented by the secretion system category, represented mainly by the *secA2* and *PPE68* genes. This was the clade with the most isolates, so that might explain the higher number of variants, all of which are nonsynonymous. However, Clade VI also presented the most clusters/subclusters compared to Clades I–V and VII. Studies suggest that the secA2 secretion system, most likely through sodA, inhibits extrinsic and intrinsic apoptosis pathways induced upon mycobacterial macrophages infection and have proposed that it prevents apoptosis and antigen-specific CD8+ cross-presentation, as well as altering the intracellular trafficking in favor of the bacteria [77].

With respect to antimicrobial resistance, *M. bovis* is intrinsically resistant to pyrazinamide (PZA) [80], so this mutation was expected in all of the genomes. The most common mutation is a CAC > GAC nonsynonymous substitution in the gene *pncA* that causes a change of histidine to aspartic acid. In this analysis, we detected a second mutation in one genome, a CTC > CGC nonsynonymous substitution that changes leucine (L) for arginine (R) in the *panD* gene [77,81]. To our knowledge, this is the only described occurrence of this mutation in a wild-type *M. bovis* strain. While it is not rare in *M. tuberculosis*, further studies targeting this mutation in *M. bovis* might provide more insight into this mechanism of resistance in this species. Similarly, isoniazid (INH) resistance, though less common, has been demonstrated from animal-recovered isolates and is a cause for concern [82,83,84,85]. INH resistance can be associated with mutations in several genes (*fabG1*, *inhA*, *iniC*, *kasA*, and *katG*); here, the five genomes with this predicted mutation involved *fabG1*. A previous study identified the *katG* mutation only among a set of 2074 genomes of *M. bovis* lineages in the Americas [85]. Another study previously performed in Catalonia also identified polyresistant *M. bovis* infection in human and sympatric sheep [86], highlighting the importance of a One Health approach in TB control to prevent the spread of TB between humans and livestock, considering the drug resistance in strains circulating among livestock.

## 5. Conclusions

Investigation of *M. bovis* strains through WGS was useful for obtaining a high-resolution overview of the population structure of this pathogen in Catalonia, which shows a heterogeneous distribution. Putative transmission clusters were identified and associated epidemiological data aided in determining explanatory factors for the transmission events, including proximity, shared pastures, and livestock-wildlife interaction, principally. Analysis SNPs within virulence genes suggests that *M. bovis* is continuously modifying specific mechanisms to maintain propagation among different hosts and environments, and antimicrobial resistance analysis highlights the importance of controlling bovine tuberculosis, as potentially resistant strains to first- and second-line drugs can infect humans. Catalonia is a region of minimal bovine TB prevalence; therefore, accuracy and high-resolution pathogen-typing are important for the identification of true sources of infection; consequently, this study complements previous research performed in the region to gain a better understanding of the pathogen’s dynamics in order to improve the eradication program.

## Figures and Tables

**Table 1:** *M. bovis* spoligotypes (SB) identified from different host types in Catalonia, Spain. Numbers in parentheses correspond to the total number of isolates from each host type. Proportion of isolates for each spoligotype is shown as a percentage (%) of the total number of isolates for that host type.

	Host Type													
	Beef (87)		Fattening (24)		Dairy (5)		Bullfighting (4)		Wild Boar (3)		Watusi (1)		Goat (1)	
	SB	%	SB	%	SB	%	SB	%	SB	%	SB	%	SB	%
**Spoligotypes**	SB0121	24.1	SB0134	25.0	SB0121	40.0	SB1685	50.0	SB0121	33.3	SB0339	100	SB0121	100
	SB0134	16.1	SB0121	20.8	SB1016	40.0	SB1095	25.0	SB0119	33.3				
	SB1337	14.9	SB0140	12.5	SB0140	20.0	SB1192	25.0	SB1337	33.3				
	SB0832	8.0	SB0120	8.3										
	SB0828	6.9	SB0832	8.3										
	SB0120	6.9	SB0124	4.2										
	SB1016	4.6	SB2312	4.2										
	SB0295	3.4	SB0130	4.2										
	SB1341	3.4	SB0142	4.2										
	SB1257	2.3	SB0295	4.2										
	SB1873	1.1	SB1259	4.2										
	SB0124	1.1												
	SB1142	1.1												
	SB0294	1.1												
	SB0152	1.1												
	SB0140	1.1												
	SB0119	1.1												
	SB0265	1.1												

**Table 2:** Defining SNP characteristics for Catalonia *M. bovis* clades obtained through WGS and SNP analysis.

Clade	Classification by [29]	Defining SNP		Product/Gene/Locus Tag	No. of Isolates	Consensus SNPs	Associated Genes *
I	9	NC_002945.4:57046	G > C	Probable conserved transmembrane protein, None, BQ2027_MB0052	11	38	*cmaA2*, *ecccb1*, *lpq*, *lipU*, *gabD2*, *mycp2*, *argS*, *gdh*, *phoH1*, *fadD36*, *dppD*, *smc*
II	29	NC_002945.4:1969776	C > T	Probable oxidoreductase, None, BQ2027_MB1780	31	5	*pks7*
III	11	NC_002945.4:3209992	C > T	Phenolpthiocerol synthesis type-I polyketide synthase, ppsA, BQ2027_MB2956	18	1	*fadD34*
IV	13	NC_002945.4:1814064	C > T	Probable integral membrane cytochrome D ubiquinol oxidase (subunit II), cydB, BQ2027_MB1648C	5	3	*eccb2*
V	14	NC_002945.4:139460	C > T	No annotated product	7	1	No annotated gene
VI	12	NC_002945.4:1254487	C > T	Probable transcriptional regulator protein, None, BQ2027_MB1160C	35	4	*eccb2*, *esxL*
VII	21	NC_002945.4:3353644	A > C	Alpha (1 → 4) glucosyltransferase, None, BQ2027_MB3058	16	96	*chaA*, *ctaB*, *dnaE1*, *esxQ*, *fadD23*, *fadD34*, *fadE22b*, *fadE24*, *fas*, *htpG*, *infB*, *kdpD*, *lipN*, *lppC*, *lppL*, *lppOb*, *ltp3*, *mdh*, *mmsA*, *mntH*, *murD*, *nadA*, *oplA*, *pca*, *pepE*, *pepR*, *pks12*, *ks8*, *PPE55a*, *PPE55b*, *PPE70*, *pptt*, *proA*, *purB*, *recC*, *rpsl*, *sigJ*, *sigL*, *thyx*, *truB*, *ugpB*, *vapc22*, *secY*, *pks6b*, *mmpL11*, *gyrA*

* Genes obtained from [26].

**Table 3:** Putative transmission clusters identified from WGS and SNP analysis of 125 *M. bovis* isolates obtained from livestock and wildlife in Catalonia, Spain, from 2008 to 2018. SNP distance is represented as an average of the SNP distances for each of the isolates involved in a cluster.

Cluster	Number of Isolates	Years of Isolation	Host Types	Average SNP Distance between Isolates	Movement Links ^1^	Type of Movement
I.1	2	2010	Dairy cattle	2	2	MIC
I.2	2	2010, 2011	Beef cattle	4	0	MIC
I.3	2	2011, 2016	Beef and dairy cattle	1	0	MIC
II.1	2	2010	Beef and fattening cattle	0	1	MIC
II.2	2	2008, 2015	Beef cattle	4	0	MIC
II.3	3	2014, 2015	Beef cattle	2.3	2	MIC
II.4	3	2009, 2011, 2016	Beef cattle	2.3	1	MIC, IOSC
II.5	13	2008, 2010, 2011	Beef and fattening cattle	5.5	4	MIC, IOSC
III.1	3	2008	Bullfighting cattle	3.7	1	MIC
III.2	3	2010, 2012	Beef and fattening cattle	5.7	2	MIC
III.3	2	2011, 2016	Beef and wild boar	2	1	MIC
V.1	5	2008, 2011, 2016	Beef cattle	7	3	MIC
VI.1	3	2010	Beef cattle	2.7	3	MIC
VI.2	2	2014	Beef cattle	1.5	1	MIC
VI.3	2	2015, 2016	Beef and wild boar	1.5	1	MIC
VI.4	3	2010-2012	Beef cattle	0.7	1	MIC
VI.5	14	2017, 2018	Beef and wild boar	4.7	1	MIC, IOSC
VI.6	2	2009	Beef and fattening cattle	0	1	MIC
VII.1	2	2009	Beef and fattening cattle	3.5	1	MIC
VII.2	3	2009	Beef cattle	3.3	2	MIC
VII.3	5	2015	Beef cattle	0.4	0	MIC

^1^ When at least two animals in a cluster shared a destination.

**Table 4:** Summary of explanatory factors for the epidemiological associations found by the identification of putative transmission clusters based on a maximum distance of 12 SNPs between *M. bovis* isolates obtained in Catalonia, Spain, from 2008 to 2018.

Most Likely Explanatory Factor	Cluster/Sub-Cluster					
Residual infection	I.1	III.1	V.1a	VI.2		
Neighborhood	V.1b	VI.4	VI.5a	VI.5b	VII.1	VII.2
Proximity	I.3	II.4	III.2			
Movement	II.1	II.3	VI.1	VI.6		
Shared pasture	II.5	VI.5	VII.3			
Livestock-wildlife interaction	III.3	VI.3	VI.5a	VI.5c		
Unknown	I.2	II.2				

**Table 5:** Antimicrobial resistance mutations associated with the *M. bovis* isolates from Catalonia.

Predicted Mutation	Nucleotide	Amino Acid Change	Resistance	No. of Isolates
*pncA* p.H57D	CAC > GAC	His57Asp	Pyrazinamide	125
*fabG1* promoter	−8T > C	Promoter mutation	Isoniazid	5

**Table 6:** Comparison of most likely causes of bovine TB breakdowns in Spanish herds.

	Guta et al., 2014	Ciaravino et al., 2021	This Study
Residual infection	22.3%	36.0%	15.0%
Wildlife interaction	13.1%	35.6%	15.0%
Introduction of cattle	5.1%	13.8%	15.0%
Sharing of pastures	7.1%	5.8%	12.0%
Contiguous spread	8.0%	3.0%	35.0%
Humans	0.3%	0.0%	ND
Goats	2.5%	0.0%	0.0%
Unknown	41.6%	5.8%	8.0%

ND: Not detected. Humans were not included as part of this study.

## Data Availability

Sequence data generated from this project is available at the NCBI Sequence Read Archive under BioProject PRJNA384785 or upon reasonable request.

## Supplementary Materials

The following are available online at https://www.mdpi.com/article/10.3390/microorganisms9081629/s1, File S1: Sequencing statistics for *M. bovis* isolates obtained from livestock and wildlife in Catalonia, Spain from 2008 to 2018, File S2: List of *M. bovis*/*M. caprae* sequences retrieved from NCBI’s Sequence Read Archive, File S3: SNP table of 5049 informative SNP sites for *M. bovis* isolates obtained from livestock and wildlife in Catalonia, Spain from 2008 to 2018, File S4: SNP analysis of Catalonia *M. bovis* isolates against a worldwide data set, File S5: Expanded global phylogenetic structure of *M. bovis* isolates from a worldwide data set, File S6: Classification and distribution of SNPs according to the functional class associated with the predicted gene annotation.
